# Validation of Depth-Averaged Flow Model Using Flat-Bottomed Benchmark Problems

**DOI:** 10.1155/2014/197539

**Published:** 2014-05-28

**Authors:** Il Won Seo, Young Do Kim, Chang Geun Song

**Affiliations:** ^1^Department of Civil and Environmental Engineering, Seoul National University, 1 Gwanak-ro, Gwanak-gu, Seoul 151-744, Republic of Korea; ^2^Department of Environmental Science and Engineering, Nakdong River Environmental Research Center, Inje University, 197 Inje-ro, Gyeongnam, Gimhae 621-749, Republic of Korea; ^3^Department of Safety Engineering, Incheon National University, 12-1 Songdo-dong, Yeonsu-gu, Incheon 406-772, Republic of Korea

## Abstract

In this study, a shallow water flow code was developed and tested against four benchmark problems of practical relevance. The results demonstrated that as the eddy viscosity increased, the velocity slope along the spanwise direction decreased, and the larger roughness coefficient induced a higher flow depth over the channel width. The mass conservation rate was determined to be 99.2%. This value was measured by the variation of the total volume of the fluid after a cylinder break. As the Re increased to 10,000 in the internal recirculating flow problem, the intensity of the primary vortex had a clear trend toward the theoretically infinite Re value of −1.886. The computed values of the supercritical flow evolved by the oblique hydraulic jump agreed well with the analytic solutions within an error bound of 0.2%. The present model adopts the nonconservative form of shallow water equations. These equations are weighted by the SU/PG scheme and integrated by a fully implicit method, which can reproduce physical problems with various properties. The model provides excellent results under various flow conditions, and the solutions of benchmark tests can present criteria for the evaluation of various algorithmic approaches.

## 1. Introduction

Modeling free surface flows in hydrodynamics, hydraulics, and environmental fluid mechanics proceeds from applying the fundamental laws of physics to a continuum. The governing equations are obtained by applying the principles of mass and momentum to a control volume of size equal to or larger than that required by the continuum hypothesis. It is usual to describe free-surface flows in a classical fluid mechanics framework using the three-dimensional Navier-Stokes equations, assuming the fluid to be Newtonian, viscous, and incompressible. Computationally, the complete resolution of the Navier-Stokes equations for a free-surface flow is known to be onerous, and the three-dimensional framework often entails numerical complexity in the meshing procedure and the implementation of the discretization method. For these reasons, when the fluid domain can be regarded as a thin layer of fluid, the two-dimensional character of a free-surface flow is enforced by a horizontal length scale considerably larger than the vertical scale and by a velocity field homogeneous over the water depth. Under these conditions, the 3D Reynolds-averaged Navier-Stokes equations can be simplified to obtain the depth-averaged shallow water equations that contain time derivative, advection terms, a surface slope term, and source terms. From a practical perspective, many cases of free-surface flows in nature can be approximated by the shallow water assumption [1–3]; thus, depth-averaged equations are generally accepted for analyzing open channel flows with reasonable accuracy and efficiency.

The most critical issue in developing a shallow water flow model is whether it can produce accurate and satisfactory results for various problems of fluid motion in shallow flow conditions. This concern becomes more important when considering that the researchers engaged in 2D horizontal (2DH) hydrodynamic modeling usually have their own individual mathematical models, spatial/temporal discretization methods, numerical schemes, algebraic solvers, and frameworks [4]. Therefore, the benchmark problems analyzed in this study were aimed to develop a more objective criterion for the evaluation of the different algorithmic approaches.

In this study, a depth-averaged hydrodynamic model adopting the Petrov-Galerkin scheme of the finite element method was developed to provide an accurate solution set for the benchmark problems of shallow water flow. The developed model features the incorporation of a secondary current effect by dispersion stresses [5], the reproduction of a convection-dominated or supercritical flow by a SU/PG test function [6], the adjustment of the internal wall velocity by the Navier-slip condition [4], and the imposition of skewed inflow velocity profiles by a beta function [7]. The first two components of the abovementioned features were employed in the benchmark test.

Among the various benchmark problems, four applications of practical relevance to the numerical modeling of shallow water flow were used to validate the shallow water flow code developed in this study. Those applications were a steady flow in a meandering channel with two curved sections: a propagating flow with cylindrical symmetry of an advancing front, an internal recirculating flow with corner eddies generated by the motion of one of the containing walls, and a supercritical flow accompanying an abruptly increased flow depth evolved by an oblique standing wave. In each problem, the accuracy of the model results was demonstrated by comparing the numerical solutions with analytic solutions, available numerical results, and/or experimental data.

## 2. Validation of the Shallow Water Flow Model

Shallow water equations model the dynamics of a shallow, incompressible, and viscous fluid to describe vertically averaged flows in three-dimensional domains in terms of horizontal velocity and depth variation. This set of equations is particularly well suited to the study and numerical simulations of a large class of geophysical phenomena, such as river flows, coastal flows, ocean circulation, or even run-off when modified with adapted source terms. In addition, the shallow water hydrodynamic model can be coupled into a transport model, in considering both flow and transport phenomena, thus making it possible to study remediation options for polluted streams and estuaries, to predict the impact of commercial projects on the environment and ecosystem and to study the allocation of allowable discharges by municipalities and by industries, in meeting water quality controls.

In the modeling of shallow water flow, there are benchmark test problems, in which analytic solutions, validated numerical results, and/or experimental data are available. Accordingly, the performance of the depth-averaged flow model can be evaluated using some measure of the difference between the numerical solution and the comparison dataset. Important features of the solution can be quantified with other reliable sources. An acceptable level of performance over a wide range of such benchmark test problems leads to confidence that a numerical scheme will perform satisfactorily for any practical problems that are not too dissimilar. Altogether benchmark test problems with known solutions are an extremely useful tool [8].

Table 1 lists the typical benchmark problems that have been studied most frequently in the shallow water flow modeling community. The aim of each benchmark test is to verify either (a) whether the depth-averaged flow model can reproduce the physical problems with various properties or (b) how accurately it produces numerical results under specific conditions. The third column of Table 1 indicates the main purpose of each test to evaluate the reproducibility of a particular flow characteristic, and the last column provides frequently cited literature references. The test problems in Table 1 include (1) a bend flow in a curved channel, (2) a discontinuity propagation evolved by the breaking of a circular cylinder, (3) an intercirculating flow formed in a side cavity, (4) a supercritical flow in a channel with a deflected wall or converging sides, (5) a combining flow in an open-channel junction, (6) an unsteady fluid motion past a bluff body in an oscillating fashion, (7) a flow over uneven bottom or flow passing through a nonprismatic channel, (8) a dam-break type flow with discontinuous initial condition, and (9) an implementation of a moving boundary scheme to capture the transition of wet and dry elements. These benchmark problems usually require a well-balanced condition, which refers to the ability of a scheme to preserve a general steady state that is satisfying the exact C-property [9]. Recent progress in well-balanced shallow water flow models in the framework of finite volume approach can be found in a surface gradient upwind method (SGUM) that integrates the source term treatment in the inviscid discretization scheme to enhance the simulation stability and accuracy for topographically varied channels [10, 11]. Hou et al. [9] proposed a slope source term treatment to transform the slope source of a cell into fluxes at its faces by splitting the integral over a cell into those of the subcells to ensure higher accuracy.

## 3. Computational Model

### 3.1. Governing Equations

The mathematical model for the calculation of depth-averaged flow is composed of the 2D shallow water equations, which are obtained by averaging the momentum and mass balance equations along the vertical direction, under several assumptions: (1) an incompressible fluid; (2) the pressure distribution in the vertical direction is hydrostatic; (3) a shallow water flow, that is, the depth-averaged values are sufficient to describe properties that vary over flow fields; (4) the bottom slopes are small in longitudinal and transverse directions; (5) barotropic, that is, any density stratification is neglected; (6) a kinematic free surface condition; (7) a no-slip bottom condition; (8) Boussinesq eddy viscosity approximation; (9) the eddy viscosity is much larger than molecular viscosity; and (10) the atmospheric pressure gradient can be ignored. The shallow water equations can be written in either conservative or nonconservative form. Then, a critical issue is which form is better to numerically describe the physical problem at hand. An ultimate answer is not available for all situations because stability, temporal, and spatial accuracy results do not change significantly between the two forms of the momentum equation [40, 41]. While the conservative form is convenient for constructing a conservative finite difference scheme and the conservation of mass and momentum can be guaranteed in the numerical solution [42], the nonconservation form yields a simpler form of the viscous terms, and in view of numerical approximations in the nonconservative form of the convective terms, an upwinding procedure is straight forward depending upon the velocity direction [43]. As Akbar and Aliabadi [44] reported, if the wave speed is much larger than the water velocity, the conservative form may become stiff, and in this case, the governing equations are usually written in nonconservative form with primitive variables. Therefore, to obtain stable solutions under various flow conditions, shallow water equations in nonconservative form are adopted in the present study.

The shallow water equations with dispersion stress terms are composed of the continuity equation and the momentum equations:

(1) $$\begin{array}{c} \frac{\partial h}{\partial t}+h\frac{\partial u_{j}}{\partial x_{j}}+u_{j}\frac{\partial h}{\partial x_{j}}=0, \end{array}$$

(2) ∂ui∂t+uj∂ui∂xj=−g∂H∂xi−g∂h∂xi +∂∂xj(ν∂ui∂xj)−gn2uiujujh4/3−∂Sij∂xj,

where *h* = flow  depth; *t* = time; *u*_*i*_, *u*_*j*_ = vertically averaged velocity component in *x*_*i*_-, and *x*_*j*_- directions, respectively, with *i*, *j* = 1,2; *g* = acceleration  of  gravity; *H* = bottom  elevation; *ν* = kinematic  eddy  viscosity; *n* = Manning's roughness coefficient; *S*_*ij*_ = dispersion stress terms arisen from the integration of the products of the discrepancy between the mean velocity and the vertically varying velocity distributions [5]. The usual boundary condition assignments to supplement the shallow water equations are the velocity postulated at the inflow boundary and the flow depth at the outflow boundary as the essential conditions. In (1)-(2), the surface shear stress due to wind is ignored because it is important only if the wind speed exceeds 100 times the flow velocity, which will not often be the case [45]. In addition, to consider the bed friction, the shear stress at the bottom is modeled using the quadratic friction formula with Manning's bottom roughness coefficient, thereby, valid in conditions with gravity-driven, fully developed open channel flows. The dispersion stress terms corresponding to the last part of (2) were activated in the benchmark test of a bend flow problem described in Section 4.1 to include the effect of secondary current more accurately. The dispersion stresses acted as a sink or source in the momentum equations, which caused the transverse convection of momentum to shift from the inner bank to the outer bank [5].

### 3.2. Numerical Model

In this study, to provide the solutions of the benchmark problem for the validation of depth-averaged flow, the SU/PG scheme of the finite element method was employed for the spatial discretization of the shallow water equations. The SU/PG scheme can be regarded as an enhanced solution method by constructing a linear combination of central and upwind difference [46–48]. The method introduces proper amount of artificial diffusion only in the flow direction, thereby providing stable and accurate results under a convection-dominated or supercritical flow condition. The scheme provides a robust approach in terms of hydrodynamic problems involving the transient flow phenomena under temporal or spatial variation and shock propagation [49] because it can accurately reflect the effect of flow acceleration by convective term and provides more stable solutions. In addition, the SU/PG scheme needs no tuning of any adjustable parameter such as artificial viscosity coefficients as other methods do. The detailed description of the discretization procedures is as follows.

The flow field *Ω* to be analyzed was divided into the subdomain *Ω* ^*e*^, *e* = 1,2,…, *n*_el_, with *n*_el_ = the number of elements. The momentum equations were discretized using either the SU/PG or the Galerkin scheme and the continuity equation using the Galerkin method. After multiplying (1) and (2) by the shape function *N*_*k*_ ∈ *H* ^1^ (*k* = 1, 2, 3 for linear triangular elements and *k* = 1, 2, 3, 4 for bilinear quadrilateral elements) from the Sobolev space *H* ^1^ of vector functions defined on the spatial domain *Ω*, and integrating over *Ω*, we applied Green's theorem to the viscous stress terms in the momentum equation to arrive at a weak formulation. Then, the finite element approximation involves finding an approximate solution in the finite dimensional subspace *H* ^*h*^ of the space *H* ^1^, by setting *u*_*i*_^*h*^ = ∑_*j*=1_^*n*^ *u*_*i*_^*j*^(*t*)*N* ^*j*^(*x*, *y*) and *h* ^*h*^ = ∑_*j*=1_^*n*^ *h* ^*j*^(*t*)*N* ^*j*^(*x*, *y*). With these statements, the problem can be cast as

(3) ∫ΩNkh(∂hh∂t+hh∂ujh∂xj+ujh∂hh∂xj)dΩ=0,∫Ω[Nkh(∂uih∂t+ujh∂uih∂xj+g∂H∂xi+g∂hh∂xi  +gn2uihujhujh(hh)4/3+∂Sij∂xj)+ν∂Nkh∂xj∂uih∂xj]dΩ  +∑e=1nel∫Ωepkh(∂uih∂t+ujh∂uih∂xj+g∂H∂xi+g∂hh∂xi   +gn2uihujhujh(hh)4/3+∂Sij∂xj)dΩe=0,

where the perturbation weighting function is defined by $p_{k}^{h}=(\overset{-}{h}\alpha /2||u^{h}||)(u_{j}^{h}(\partial N_{k}/\partial x_{j}))$ with ||**u** ^*h*^|| = the Euclidian norm of velocity; *α* = coth⁡(*γ*/2) − (2/*γ*) is the quadrature points; and $\gamma =||u^{h}||\overset{-}{h}/\nu$ is the element Reynolds number. The element characteristic length $\overset{-}{h}$ is given by [50]

(4) $$\begin{array}{c} \overset{-}{h}=\frac{1}{||u^{h}||}\left(\left|h_{1}\right|+\left|h_{2}\right|\right), \end{array}$$

where *h*_1_ = *a*_*j*_ *u*_*j*_ and *h*_2_ = *b*_*j*_ *u*_*j*_ with *j* = 1, 2; *a*_*j*_ and *b*_*j*_ are the components that join the midpoint of opposite sides. Hence, *h*_1_ and *h*_2_ are the projections of *u*_*j*_ in the direction of flow. Consequently, the SU/PG scheme includes the weight function perturbed by the production of a velocity and gradient of shape function, which only acts in the flow direction. This effect decreases the instability of nonlinear convective acceleration in the momentum equations.

Formulation (3) was time-discretized with the fully implicit method to achieve solution robustness and efficiency because explicit schemes can be highly restrictive in the presence of supercritical wave propagation.

There are no restrictive compatibility conditions, such as the LBB (Ladyzenskaja-Babuška-Brezzi) condition [42, 51, 52] on the discrete spaces; thus piecewise bilinear interpolations were used for all fields in the computations reported here, and three-point Gaussian quadrature was employed for numerical integration [4]. Bilinear quadrilateral elements (*i*, *j* = 1, 2, 3, 4) with nonorthogonal edges were transformed to straight-sided orthogonal elements by introducing the natural coordinates *ξ* = *ξ*(*x*, *y*) and *η* = *η*(*x*, *y*). The shape functions *N*_*i*_ in the natural coordinate system are defined as

(5) $$\begin{array}{c} N_{i}=\frac{\left(1+\xi_{i}\xi \right)\left(1+\eta_{i}\eta \right)}{4},\;i=1,2,3,4, \end{array}$$

where *ξ*_*i*_ and *η*_*i*_ are corner nodes of the square stretching from (−1, −1) to (1, 1).

The nonlinear system resulting from the finite element discretization of the flow equations is solved using the Newton-Raphson method, and the linear set of equations was solved by the multiple frontal method to save the storage of the entire assembled matrix by an interleaving assembly and elimination operation [53]. At any time step, the model was run until a steady state was achieved, which was determined by a relative depth convergence *R*, which is given below:

(6) $$\begin{array}{c} R=\sqrt{\frac{\sum {\left(h_{i}^{n+1}-h_{i}^{n}\right)}^{2}}{\sum {\left(h_{i}^{n}\right)}^{2}}}\leq 1.0\times 10^{-5}, \end{array}$$

where *h*_*i*_^*n*^ and *h*_*i*_^*n*+1^ are the local water depths at the *i*th node at time step *n* and *n* + 1, respectively.

## 4. Benchmark Problems

Among the nine problems included in Table 1, some require extensive analysis and thus need to be addressed in separate articles. Consequently, the first four cases of practical relevance in Table 1 were considered in this study as benchmark problems for the validation of the depth-averaged flow model. Although all the flow problems were solved in flat bottoms and the influence of the bed slope change is not considered, the benchmark tests include various circumstances for the fluid motion, and different combinations of initial/boundary conditions as shown in Table 2. For each benchmark simulation, structured quadrilateral meshes were employed, and the grid independency was checked by more than three mesh layouts. Accordingly, all the numerical results reported herein are converged solutions with no influence from the mesh configuration.

### 4.1. Steady Problem

**Figure 1:**
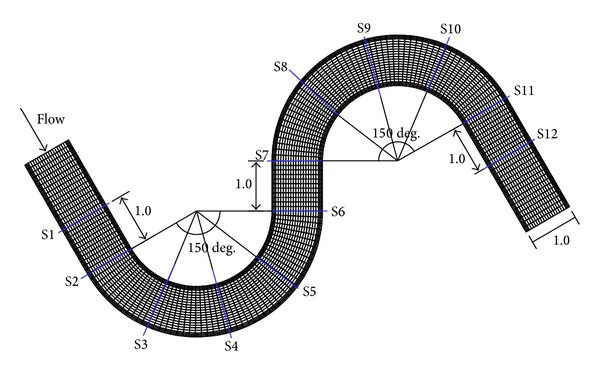
Mesh layout and measured sections for the simulation of the meandering channel (unit: m).

A meandering channel is a suitable geometry to test a new flow modeling code because accelerated velocity and superelevation of the water surface by centrifugal force are usually observed at the bends. Therefore, a steady numerical simulation was conducted to analyze the transverse velocity distributions at the apex of the bends. As long as boundary conditions are imposed in a manner which leads to a well-posed steady solution, the unsteady computations with constant boundary conditions will eventually converge to a steady solution that is obtained by excluding the time-derivative terms in (1) and (2). We confirmed this fact, and the numerical results by the steady solver are reported in this section.  Figure 1 shows the mesh layout and channel information. It has 4,080 nodes and 3,898 quadrilateral elements with a rectangular cross-section and zero bottom slope. S4 and S9 are the locations of the apices of the first and second bends. The channel width is 1.0 m, and the sinuosity is 1.52. Seo and Park [54] measured three-dimensional velocity structures with a micro-ADV at the 12 sections. In this study, 3 cases out of 12 hydraulic measurements were provided, to present the measured velocity data.

**Figure 2:**
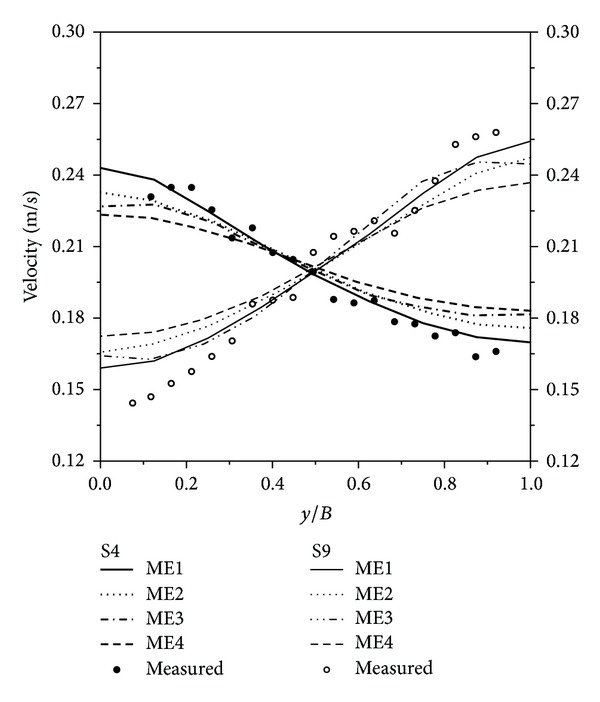
Velocity distributions of the meandering channel flow according to varying eddy viscosities.

**Figure 3:**
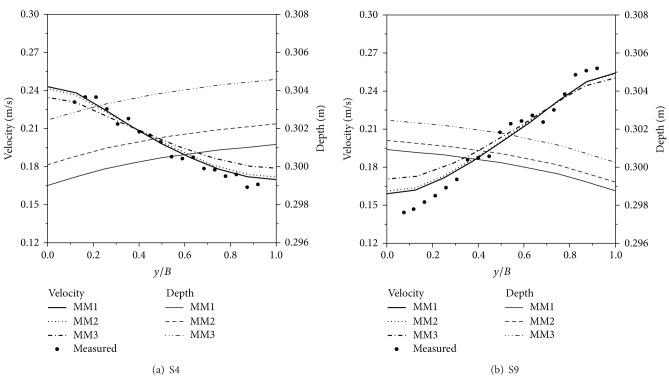
Velocity and depth distributions of the meandering channel flow according to varying Manning coefficients.

To determine the eddy viscosity and Manning coefficient, simulations were performed under the conditions given in Table 3(a). Four eddy viscosity coefficients and three Manning coefficients were considered, and computational results are plotted in Figures 2 and 3.  Figure 2 shows the velocity distribution according to varying eddy viscosities. As the eddy viscosity increased, the slope became milder, and the velocity difference between the left and right bank decreased because the increased eddy viscosity coefficient diffused the velocity field, by reducing the velocity gradient between neighboring points. Although the measured velocities are oscillating, the mean lines matched well with ME1. Thus, eddy viscosity was adopted as 10^−3^ m^2^/s for every direction.  Figure 3 shows the velocity distribution according to varying Manning coefficients. The measured velocity data matched well with MM1, in the average sense. Regarding the water depth, MM2 and MM3 gave inappropriate results because they had higher depths along the transverse direction than the assigned freestream depth. In contrast, MM1 yielded reasonable results, in terms of the superelevation phenomena as well as velocity distribution. On these grounds, the Manning coefficient was selected as 0.013, which agrees with the suggested roughness coefficient for a smooth steel surface [55].

**Figure 4:**
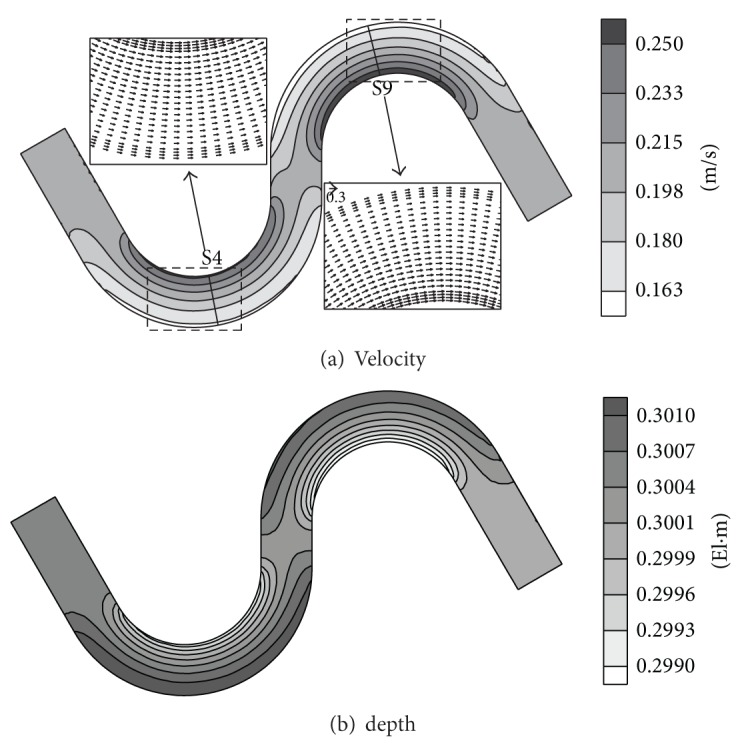
Velocity field and depth contour for M1 in meandering channel flow.

**Figure 5:**
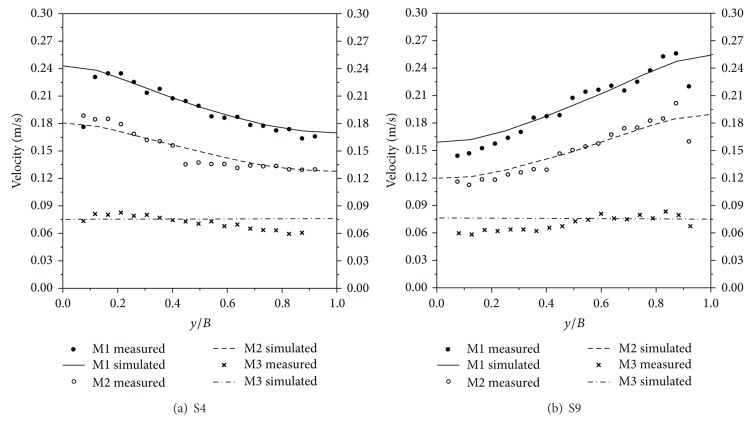
Comparison of transverse velocity distributions for meandering channel flow at the apices of the bends.

With these parameters, three additional simulations were performed under the conditions provided in Table 3(b). The upstream and downstream boundary conditions were assigned the same values as the experimental conditions of Seo and Park [54]. The ratio of depth to radius of curvature used in dispersion stress to include the effect of the secondary current was 0.2. A detailed description regarding the dispersion stress and input parameters can be found in Song et al. [5]. The velocity field and water depth for the M1 case are illustrated in Figure 4. Uniform flow developed up to the straight section. As the flow passed through S2, it was accelerated by the centrifugal force, and the velocities at the inner bank were faster than those of the outer bank, and again retrieved uniform velocity distribution at the straight part, located between S6 and S7. From the magnified views of the S4 and S9 sections, it is observed that the peak velocity of the primary flow is located near the inner bank, at the apices of the bends. In accordance with the velocity distribution, weak superelevation occurred, as shown in Figure 4(b).  Figure 5 shows a comparison of the computational results with the measured data at two apices of the bends S4 and S9. The two results are in good agreement, in all cases. The maximum velocity at the second bend was approximately 40% higher than that of the first bend. However, in the case of M3, the velocity values were constant across the spanwise direction. This is attributed to the low velocity imposition at the inflow boundary, which disabled the flow acceleration at the bends.

### 4.2. Symmetrical Property and Mass Conservation

The breaking of a dam having a cylindrical geometry presents the check of model performance to conserve symmetry because this problem is essentially one-dimensional in the radial direction, when transformed into polar coordinates. Although there is no exact solution to be compared with [16], this problem provides the time evolution of the subsequent waves, and the model's ability to capture shocks can be evaluated. In addition, the conservation of mass can be tested indirectly since the initial volume of two regions of still water, separated by a cylindrical wall, should be exactly the same as the volume after steady state.

**Figure 6:**
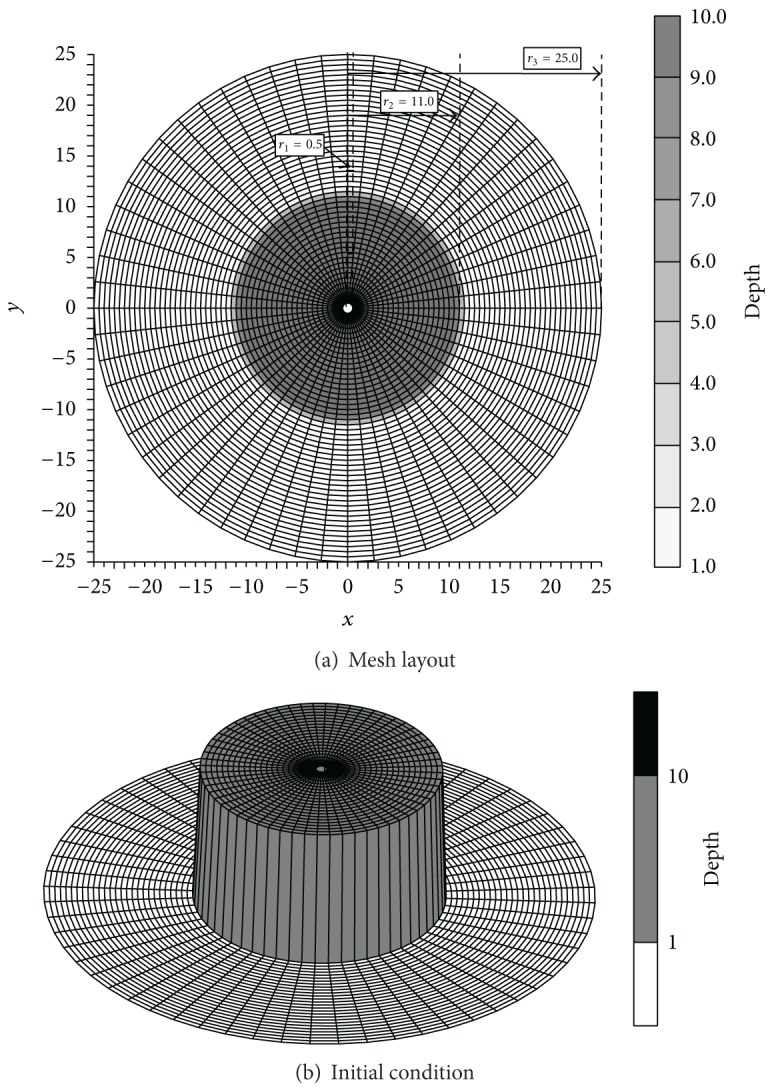
Mesh layout and initial condition for cylinder breaking simulation (unit of depth: m).

The computational mesh used for this benchmark problem consists of 50 elements of 0.5 m streamwise length in the radial direction, and 60 elements in the circumferential direction, as shown in Figure 6(a). A cylindrical water column of radius 11 m and height 10 m, which was initially at rest, was allowed to collapse instantaneously under gravity, into a region of still water of radius 25 m and height 1 m (Figure 6(b)). In this example, the conventional boundary conditions, represented by the discharge at the upstream boundary, and the water surface at the downstream boundary can be evaded, thereby assigning inner and outer boundaries as just impermeable or solid walls as shown in Table 2. The computational time step was 0.01 sec, and Manning coefficient of 0.010 and eddy viscosity of 0.003 m^2^/sec were adopted to provide stable and accurate solutions.

**Figure 7:**
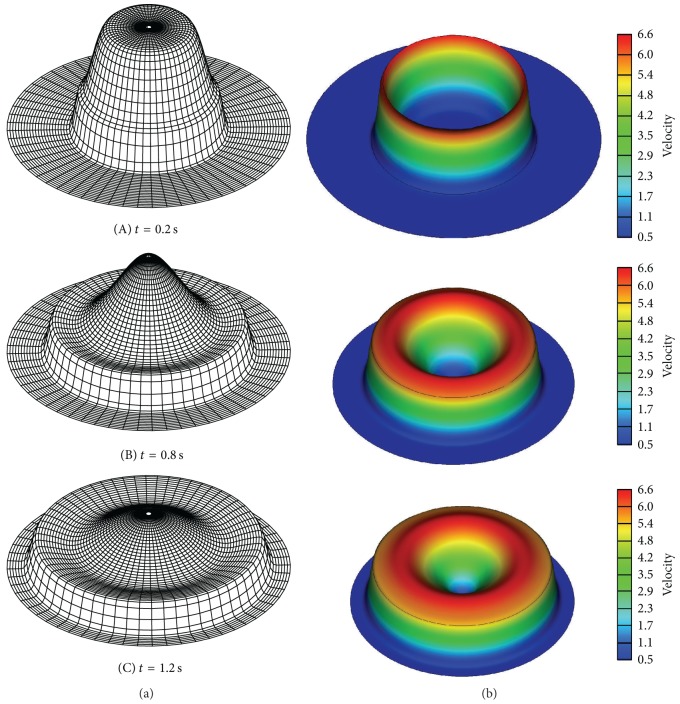
Evolution of water surface (a) and velocity (b) after breaking of the circular cylinder (unit of velocity: m/s).

The numerical results of water surface evolution and velocity configuration after the breaking of the circular cylinder are shown in Figure 7. The results were plotted before the diverging bore reached the outer boundaries of the domain. The simulated results showed that there was an outward-propagating circular shock wave and an inward-propagating circular rarefaction wave. Both the advancing front and the depression wave preserved cylindrical symmetry, and the present model resolved the bore wave without oscillations. The surface elevation near the center decreased smoothly, while the outside shock wave continued to propagate outward, with intense strength.

**Figure 8:**
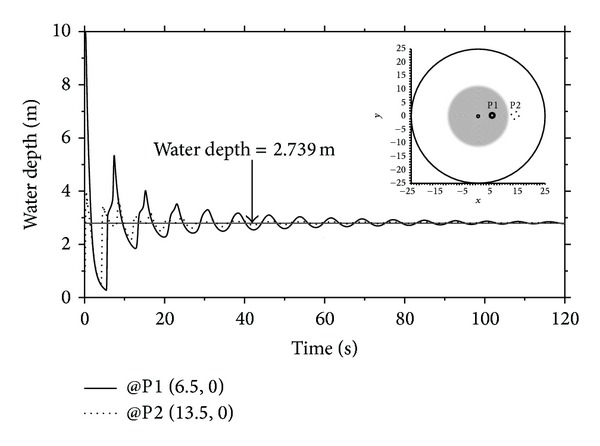
Depth convergence histories at two points after breaking of the circular cylinder.

The total volume of the fluid should be preserved at any time during simulation to ensure conservation of mass. The fluid volume is closely related to the flow depth, and the flow depth would finally converge to a specific value, after enough time elapses. Thus, examining the time variation of the water depth is important, to check the mass conservation. The steady-state depth after the completion of breaking of the cylinder could be easily calculated as *h* = 2.739 m.  Figure 8 shows the depth convergence histories at two points: P1 (6.5, 0) placed inside the discontinuity plane, and P2 (13.5, 0) in the outer. The lowest values of the water depth at the inside and outside points were 0.272 m and 0.492 m, respectively, which was smaller than the initial water depth of the outer basin. This clearly demonstrated the propagation of a depression wave. After experiencing irregular oscillations during the initial stage, the flow depths approached to the value of 2.761 m, and the mass conservation rate in terms of the total volume of the fluid was 99.2%. In general, errors in finite element method are attributed to the physical modeling error, the discretization error, and the numerical error [56, 57]. In addition to the physical modeling error from which no numerical scheme can be free, the discretization error arising from the mesh generation and the numerical error resulting from the Gaussian quadrature formula and the round-off error are difficult to avoid. Among these error sources, mesh size and finite digit arithmetic to represent numbers are considered as the main causes of the mass conservation error, 0.8%. This error can be reduced by element refinement or using high-precision floating-point computation.

### 4.3. Vortex Representation

Internal recirculating flows generated by the motion of one of the containing walls have served as a model problem and possess practical importance, since they display almost all flow characteristics, such as vortex configurations, corner eddies of different size and shape, boundary layers, and various instabilities associated with the transition of turbulence in the simplest geometry [58]. Most of the existing literature on 2D cavity flows dealt with a rectangular cavity, in which the flow is generated by the steady, uniform motion of one of the walls alone, for example, the lid. The essential feature of the test problem is to predict the location and the intensity of various vortices inside the cavity. For a moderately high Reynolds number, an abundant literature is available for this flow problem. The linear stability analysis by Bruneau and Saad [21] and Das and Kanna [59] reported that the critical Re for the 2D lid-driven cavity problem, when the steady solution loses its stability to the benefit of a periodic solution, which corresponds to the localization of the first Hopf bifurcation, is Re = 8,000. Beyond this Re, the flow in a two-dimensional-driven cavity is unsteady; therefore, a steady solution does not exist; hence a steady solution at high Reynolds numbers is not computable [60].

In the analysis of the lid-driven cavity problem, the stream function-vorticity formulation allows the elimination of pressure from the problem and automatically satisfies the continuity constraint. On the other hand, the value of the vorticity at no-slip boundaries is difficult to specify, and a poor evaluation of this boundary condition leads, almost invariably, to serious difficulties in obtaining a converged solution [61, 62]. Hence, the primitive variable formulation is the preferred route to the solution of complex fluid flows in engineering geometries. If the no-slip and impermeability conditions are applied to the side walls, there will be a discontinuity in the boundary conditions at the two top corners, where the side walls meet the lid. This is the origin of the so-called corner singularity, which is of theoretical interest, but which only plays a minor role in the overall field [63, 64].

In this section, the square cavity problem is examined. The computational mesh used in the simulation had 6,561 nodes and 6,400 quadrilateral elements. The Bubnov-Galerkin scheme with parameters of *ν* = 10^−4^ m^2^/s and *n* = 0.013 was employed, since there is no upwind direction of the velocities in a driven cavity flow, and the SU/PG scheme would overdiffuse the vortex formation. The Reynolds number chosen for this cavity problem is based on the lid velocity, the width of the cavity, and the kinematic viscosity of the working fluid. The boundary conditions were of the Dirichlet type for velocities, the usual impermeability and no-slip conditions along the stationary walls, and the initial conditions correspond to a quiescent fluid. All calculations were initiated from zero velocity, without using information from previous solutions at lower Reynolds numbers. To facilitate the discussion of the eddies, they were designated as BR1, BR2, BL1, TL1, in which the abbreviations refer to bottom right, bottom left, and top left corners of the cavity, respectively. The number following these abbreviations refers to the vortices that appear in the flow, which are numbered according to the eddy size.

**Figure 9:**
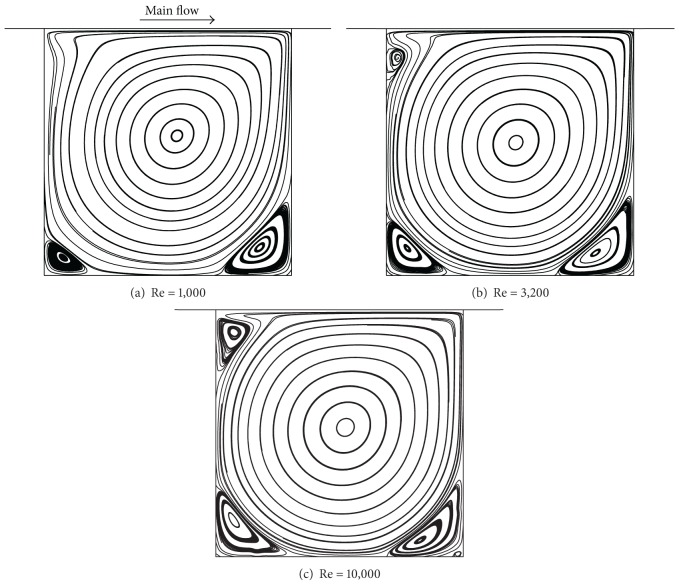
Streamline configuration of side cavity problem by Re.

**Figure 10:**
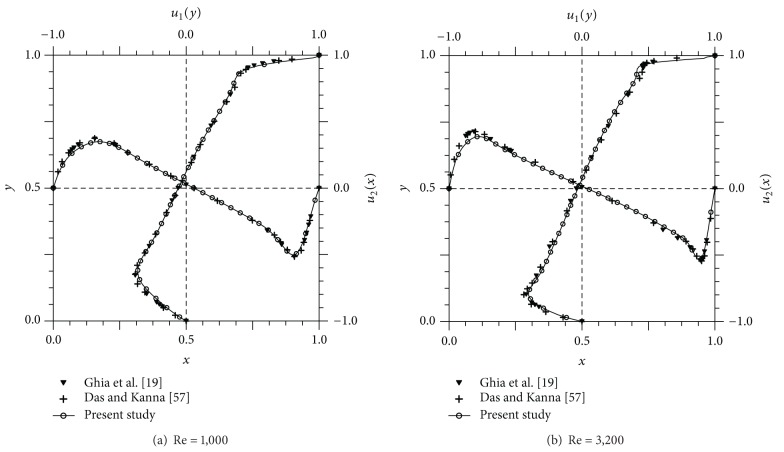
Velocity profiles of side cavity problem along the horizontal and vertical center lines (unit of velocity: m/s).

**Figure 11:**
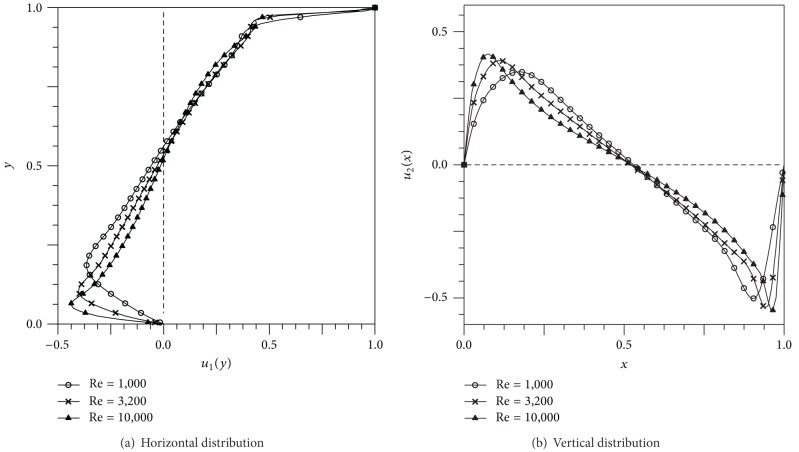
Velocity distributions of side cavity problem along the mid-sectional lines (unit of velocity: m/s).

The streamline configurations for the square side-cavity flow configurations with Re = 1000, 3200, and 10000 are shown in Figure 9. As the Re increased, the primary vortex shifted to the center, and more corner secondary vortices were formed. With increase of Re, the secondary vortices were also convected towards the center of the domain, and the size of the vortices at the corners increased. The secondary vortices of BR2 were evolved at Re = 10,000. In Figure 10, the velocity profiles along the horizontal and vertical lines passing through the geometric center of the cavity are compared with Das and Kanna [59] and Ghia et al. [19], which is the most frequently referenced source of results on the driven cavity flow. It exhibited excellent agreements with the results of other authors. To represent the variation of flow configuration according to the Re, the velocity distributions along the mid-sectional lines through the cavity center for three Re are illustrated in Figure 11. As the Reynolds number increased, the extremal values of the velocity components increased in magnitude and the turning points became progressively closer to the wall. This phenomenon indicates that the thinning of the wall boundary layers with an increase in Re is evident from these profiles, as noted by Ghia et al. [19]. A similar behavior was observed for the vertical component in Figure 11(b). The vorticity intensity of the primary and secondary vortices, calculated by *ω* ≡ ∂*u*_2_/∂*x*_1_ − ∂*u*_1_/∂*x*_2_, is provided in Table 4. The values for the central (primary) vortex were in excellent agreement with earlier results. Some differences were found in the bottom corner values. There are many aspects which could account for this wide range of results: accuracy/convergence rate of the method employed and issues such as upwinding, use of primitive variable or stream function-vorticity formulation, convergence criteria, and arithmetic precision. As the Re increased to 10000, there was a clear trend towards the theoretical infinite Re value of −1.886, suggested by Burggraf [65].

### 4.4. Reproduction of Supercritical Flow

When a supercritical flow passes through a channel with decreasing width, the deflected channel wall generates an oblique standing wave accompanying an abruptly increased flow depth, which is induced by means of an interaction between a supercritical flow and a wall constriction. This phenomenon is known as an oblique hydraulic jump. It has been considered to confirm the capability of handling high-speed discontinuous flows and the stability under a supercritical flow situation.

**Figure 12:**
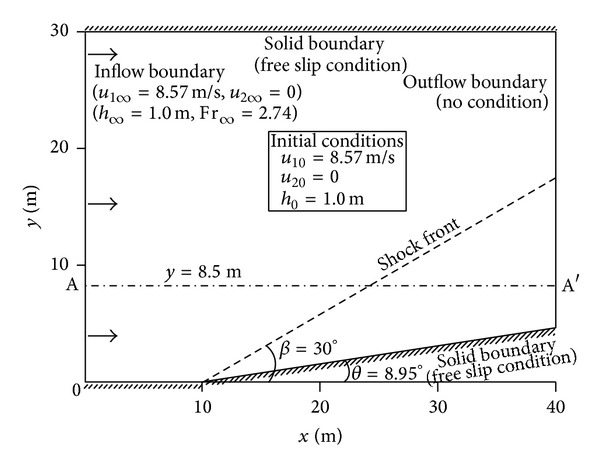
Schematic diagram and simulation conditions of oblique hydraulic jump problem in converging channel (plan view).

The computational domain and simulation conditions are shown in Figure 12. The inflow boundary conditions for assigning supercritical flow are *u*_1*∞*_ = 8.57 m/s (longitudinal velocity value at upstream boundary), *u*_2*∞*_ = 0 m/s (transverse velocity value at upstream boundary), and *h*_*∞*_ = 1.0 m (water depth at upstream boundary); this corresponds to Fr = 2.74 at the inflow boundary. No boundary conditions are required on the outflow section, since the flow is supercritical. In addition, free-slip conditions are imposed on the side walls, and the initial conditions are identical to the upstream boundary conditions. Finite elements made up of 4,800 (80 × 60) rectangular elements and parameters of *n* = 0.01, and *ν* = 0.3 m^2^/s were used to reproduce the discontinuous flow in a nonprismatic channel with zero bed slope.

**Figure 13:**
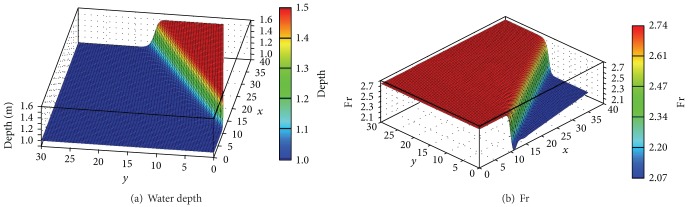
Formation of water depth and Froude number after oblique hydraulic jump.

Figure 13 shows the distribution of water depth and Fr number after the shock is completely developed. The results were obtained without considering the air entrainment since the interaction between the free surface and the air is not the target of present research. The shock was well captured within the span of a few elements, and the angle formed by the oblique hydraulic jump was sharply reproduced. Accordingly, the discontinuous water surface and corresponding Fr number devoid of oscillations were obtained.

The analytical expressions for the oblique hydraulic jump can be found in Chow [55] as follows. The exact solutions for flow velocity (||**u**_*s*_||), water depth (*h*_*s*_) inside the shock, and the angle (*β*) between the shock line and* x*-axis can be expressed by the following equations:

(7) tanθ=tanβ(1+8Fr∞2sin2β−3)2tan2β+1+8Fr∞2sin2β−1,hsh∞=12(1+8Fr∞2sin2β−1),||us||||u∞||=cos⁡β1+tan2(β−θ).

According to the above equations, the analytical solutions are *h*_*s*_ = 1.5 m, and ||**u**_*s*_|| = 7.956 m/s, which result in Fr_*s*_ = 2.075. The angle formed by the oblique hydraulic jump is *β* = 30°.  Table 5 lists a comparison of the analytical and numerical values, including the average value of the velocity magnitude, the water depth, and the Fr number on the shock side, the minimum depth below the shock, and the shock angle. The computed angle of the wave front is *β* = 30.06°, and the flow depth and the resultant velocity across the jump are 1.500 m and 7.966 m/s, respectively. These values agreed well with the analytical solutions given by (7), within the error bound of 0.20%. It is clear that the present model showed satisfactory predictions, compared with other models of Levin et al. [66] and Ricchiuto et al. [67]. The reasons for the relatively good performance of the present model are as follows. Levin et al. [66] implemented explicit time integration using the Adams-Bashforth scheme, which is sensitive to the selection of a time step and start-up method under high Froude number flow condition. Ricchiuto et al. [67] used a conservative form of the shallow water equations without a viscous stress term so that the energy transfer as well as the transport of hydraulic properties of supercritical flow needed to be checked closely. On the contrary, the present model adopting the nonconservative form of shallow water equations weighted by SU/PG scheme and integrated by fully implicit method gave excellent results under high Re flow condition.

**Figure 14:**
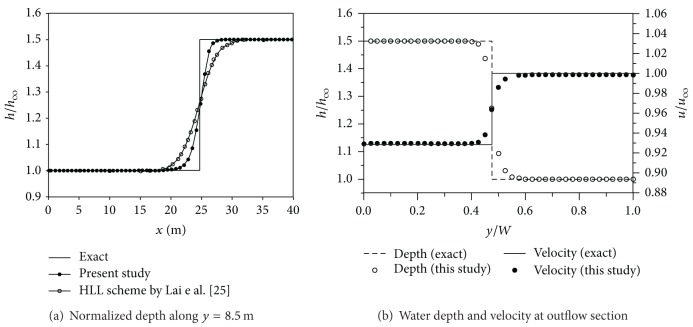
Comparison of water depth and velocity for oblique hydraulic jump problem.

The water depth along a longitudinal line and the water depth/velocity distributions at the outflow section are illustrated in Figure 14. All the values were normalized by the flow variables at the inflow boundary.  Figure 14(a) shows comparisons of the exact solutions with the simulated water depth profile along line AA′ marked as the dash-dot line in Figure 12. As shown in the figure, the present model resolved the shock front more sharply than the HLL scheme by Lai et al. [25].  Figure 14(b) displays a comparison of the water depth and velocity at the downstream boundary. The exact water depth and velocity values are plotted by the dashed and solid line, respectively; the hollow circle denotes the depth prediction result, and the solid circle means the numerical value of velocity. Both of the numerical results provided good approximations to the exact solutions, over the width of the outflow section.

## 5. Conclusions

A shallow water flow code adopting the SU/PG scheme of the finite element method was developed to solve flat-bottomed benchmark problems for depth-averaged flow modeling. Four tests were implemented in the flow problems and the channel geometries, such as a meandering channel, a breaking of a circular cylinder, a square side-cavity, and an oblique hydraulic jump.

In the meandering channel simulation, the accelerated velocity and superelevation activated by the centrifugal force at the bends were clearly presented and compared with experimental data. As the eddy viscosity increased, the velocity slope along the spanwise direction decreased and the larger roughness coefficient induced a greater flow depth over the channel width. The breaking of a circular cylinder problem was considered to check the mass conservation, as well as the preservation of cylindrical symmetry of the advancing front and the depression wave. The present model resolved the bore wave without oscillations, and the mass conservation rate in terms of the total volume of the fluid was 99.2%. The representation of eddies and predictions of the primary and secondary vortex intensity were provided in the square side-cavity simulation. With an increase of the Re, the primary vortex shifted to the center, and the secondary vortices were also shifted toward the center of the domain, and the size of the vortices at the corners increased. As the Re increased to 10,000, the intensity of primary vortex had a clear trend towards the theoretical infinite Re value of −1.886. Finally, the resolution of the deflected standing wave accompanying abruptly increased flow depth was presented to determine the capability of supercritical flow simulation. The computed values agreed well with the analytic solutions, within the error bound of 0.2%. The present model adopting the nonconservative form of shallow water equations weighted by the SU/PG scheme, and integration by a fully implicit method provided excellent results under the flow condition of a high Froude number.

The benchmark tests considered in this study include various circumstances of fluid motion, and different combinations of initial/boundary conditions can serve as performance evaluation criteria for any shallow water flow models.

## Figures and Tables

**Table 1:** Benchmark problems of shallow water flow.

Benchmark problems	Geometry	Main concerns and issues	Frequently referred sources for comparison or recent contributions
Bend flow	Meandering channel	(i) Velocity acceleration by centrifugal force (ii) Superelevation of water surface level (iii) Secondary current	(i) Rozovskii [12] (ii) de Vriend [13] (iii) Odgaard [14] (iv) Seo et al. [15]

Discontinuity propagation	Circular cylinder	(i) Shock-capturing property (ii) Time evolution of subsequent wave (iii) Preservation of symmetry (iv) Mass conservation (v) Test for lack of oscillations	(i) Alcrudo and Garcia-Navarro [16] (ii) Mingham and Causon [17] (iii) Erduran et al. [18]

Side cavity	Side pocket with square domain	(i) Generation of corner vortex (ii) Intensity of vortices according to Re (iii) Velocity profiles along the center lines	(i) Ghia et al. [19] (ii) Botella and Peyret [20] (iii) Bruneau and Saad [21]

Supercritical flow	(i) A channel with deflected wall (ii) Converging channel	(i) Reproduction of oblique standing wave (ii) Resolution of discontinuity without oscillation (iii) Quantitative comparisons with analytic solutions	(i) Ippen and Dawson [22] (ii) Zhao et al. [23] (iii) Caleffi et al. [24] (iv) Lai et al. [25]

Merging flow	Confluent channel	(i) Magnitude of contracted flow (ii) Range of recirculation zone (iii) Flow behavior by junction angle	(i) Weber et al. [26] (ii) Liu et al. [27] (iii) Thanh et al. [28] (iv) Song et al. [5]

External flow	Rectangular channel with airfoil or cylinder(s)	(i) Reduction of drag force (ii) St number of vortex street under low Re (iii) Analysis of bow wave and separation angle	(i) Dennis and Chang [29] (ii) Braza et al. [30] (iii) Yulistiyanto et al. [31] (iv) Seo and Song [4]

Flow over uneven bottom or through nonprismatic channel	A channel with bump/weir or continuously varying width	(i) Treatment of source terms (ii) Flow characteristic near transition (iii) Quantitative comparisons with analytic solutions	(i) Bermudez and Vazquez [32] (ii) MacDonald [8] (iii) Zhou et al. [33]

Transcritical flow	Rectangular domain with discontinuous initial condition	(i) Check for proper imposition of boundary conditions (ii) Flow over wet/dry bed (iii) Shock-capturing property (iv) Formation of the advancing front and the depressive wave (v) Test for lack of oscillations	(i) Katopodes and Strelkoff [34] (ii) Fennema and Chaudhry [35] (iii) Fraccarollo and Toro [36]

Wet/dry	(i) A channel with linearly varying bottom slope (ii) Parabola basin	(i) Capturing the advancing/receding wetting front (ii) Mass conservation (iii) Extension to flood inundation model	(i) Thacker [37] (ii) Balzano [38] (iii) Oey [39]

**Table 2:** Initial and boundary conditions for benchmark test problems considered in this study.

Test	Initial conditions		Boundary conditions			
	Velocity	Depth	Inflow	Wall		Outflow
				Stationary	Motional	
Meandering channel	Zero	Constant	Subcritical	Slip conditions	—	Water surface
Breaking of a circular cylinder	Zero	Discontinuous	—	Slip conditions	—	—
Square side-cavity	Zero	Constant	—	No slip conditions	Velocity imposition parallel to wall	—
Oblique hydraulic jump	Constant	Constant	Super-critical	Slip conditions	—	—

**Table:** (a) Parameters determination

Case	Inflow BC Q (cms)	Outflow BC h (El·m)	*ν*_*xx*_ (m^2^/s)	*ν*_*xy*_ (m^2^/s)	*ν*_*yy*_ (m^2^/s)	*n*
ME1	0.06	0.30	10^−3^	10^−3^	10^−3^	0.013
ME2			10^−3^	5 × 10^−3^	10^−3^	
ME3			5 × 10^−3^	10^−3^	5 × 10^−3^	
ME4			5 × 10^−3^	5 × 10^−3^	5 × 10^−3^	
MM1			10^−3^	10^−3^	10^−3^	0.013
MM2						0.025
MM3						0.050

**Table:** (b) Simulation cases

Case	Inflow BC Q (cms)	Outflow BC h (El·m)	*ν*_*xx*_ (m^2^/s)	*ν*_*xy*_ (m^2^/s)	*ν*_*yy*_ (m^2^/s)	n
M1	0.06	0.30				
M2	0.06	0.40	10^−3^			0.013
M3	0.03	0.40				

**Table:** (a) *Re* = 1,000

Vortex	Erturk [60]	Bruneau and Saad [21]	Spotz [64]	Botella and Peyret [20]	Ghia et al. [19]	Present study
PV	−2.067	−2.067	−2.053	−2.067	−2.050	−2.067
BR1	1.111	1.112	1.044	1.155	1.155	1.110
BL1	0.350	0.362	0.239	0.352	0.362	0.354

**Table:** (b) *Re* = 10,000

Vortex	Erturk [60]	Ghia et al. [19]	Present study
PV	−1.910	−1.881	−1.879
BR1	3.752	4.053	3.728
BR2	0.302	0.313	0.301
BL1	2.146	2.086	2.136
TL1	2.297	2.183	2.273

**Table 5:** Comparison of numerical results by oblique hydraulic jump.

Results		||**u**_*s*_|| (m/s)	*h*_*s*_ (m)	Fr_*s*_	*h*_min⁡_ (m)	*β* (°)
Exact values		7.956	1.500	2.075	1.000	30

Levin et al. [66]	Values	7.633	1.623	2.024	0.932	—
	Error (%)	4.06	8.20	2.46	6.80	—

Ricchiuto et al. [67]	Values	7.931	1.511	2.060	0.9997	—
	Error (%)	0.31	0.73	0.72	0.03	

This study	Values	7.966	1.500	2.077	1.002	30.06
	Error (%)	0.13	0.00	0.10	0.20	0.20
